# Rethinking one of criminology’s ‘brute facts’: The age–crime curve and the crime drop in Scotland

**DOI:** 10.1177/1477370817731706

**Published:** 2017-11-24

**Authors:** Ben Matthews, Jon Minton

**Affiliations:** University of Edinburgh, UK; University of Glasgow, UK

**Keywords:** Age–crime curve, crime drop, data visualization, Scotland, sex differences

## Abstract

Examining annual variation in the age–crime curve as a way to better understand the recent crime drop, this paper explores how the age distribution of convicted offending changed for men and women in Scotland between 1989 and 2011. This analysis employs shaded contour plots as a method of visualizing annual change in the age–crime curve. Similar to recent findings from the USA, we observed falling rates of convicted offending for young people, primarily owing to lower rates of convicted offending for young men. In contrast to the US literature we also find increases in the rate of convicted offending for those in their mid-twenties to mid-forties, which are relatively greater for women than men. Analysis of annual change shows different phases in the progression of these trends, with falls in prevalence during the 1990s reflecting lower rates of convictions for acquisitive crime, but falls between 2007 and 2011 being spread across multiple crime types. Explanations of the crime drop in Scotland and elsewhere must be able to account for different patterns of change across age, sex, crime type and time.

## Introduction

The age–crime curve (ACC) has a long history in criminology. First described in the 1830s by Adolphe Quetelet (2003 [1831]), this relationship has been characterized as ‘one of the brute facts of criminology’ (Hirschi and Gottfredson, 1983: 555). However, recent years have seen a number of new analyses of change in the ACC (Blumstein, 2006; Cook and Laub, 2002; Farrell et al., 2015; Kim et al., 2015) that represent a different approach to the study of the ACC, using change in this distribution as a way to unpick trends in macro-level crime rates. This contrasts with previous work, which was primarily concerned with the description and explanation of the micro-level relationship between a person’s age and propensity to offend (for example, Greenberg, 1985; Hirschi and Gottfredson, 1983). Change in the aggregate ACC has been of particular recent interest to identify whether the recent crime drop (Van Dijk and Tseloni, 2012) reflects declining crime rates for all ages, or whether there are different patterns of change for different age groups.

This emerging body of work has some important omissions. First, it primarily focused on the USA, and currently it is not known whether findings from US studies are replicated elsewhere. Second, to our knowledge no studies have analysed recent change in the ACC for men and women separately, an important limitation given the typical sex differences in the ACC (Farrington, 1986). Third, these recent studies have analysed age–crime curves from disparate time points rather than annual variations in this distribution, throwing away potentially useful information about change in the ACC over shorter intervals. This paper aims to fill these three gaps in this new body of work on the age–crime curve by analysing annual change in the ACC for men and women over the course of the recent crime drop in Scotland. This analysis finds substantial change in the aggregate ACC over this period primarily owing to declines in youth convictions, and with different patterns of change between men and women, during different periods and between different types of crime.

## Literature review

### The ‘great debate’: Is the age–crime curve variant or invariant?

**Figure 1.**
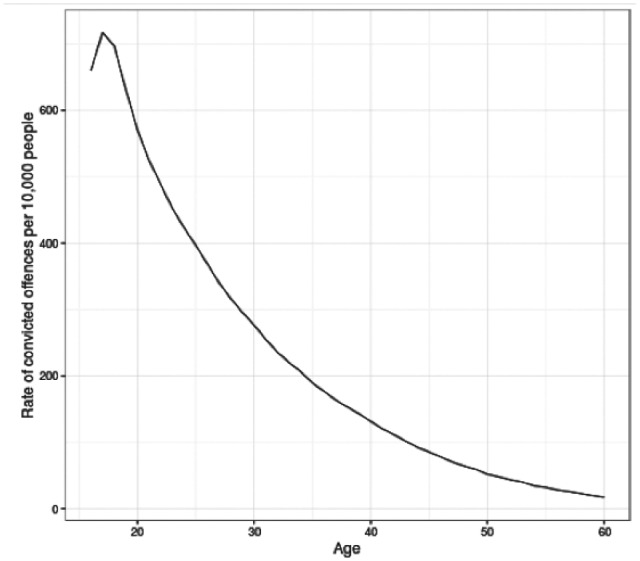
Age–crime curve for all convicted offences in the Scottish Offenders Index, 1989–2011.

The familiar shape of the age–crime curve is one of the most consistently observed empirical findings in criminology (Hirschi and Gottfredson, 1983). This curve has an ‘asymmetrical bell shape’ (see Figure 1), showing that the proportion of people who offend increases through adolescence, peaks in the teenage years and then declines from the late teens or early twenties (Loeber and Farrington, 2014: 12). The 1980s saw heated debate as to whether the age–crime curve should be considered universally invariant. On one side of this debate, Gottfredson and Hirschi contended that the ACC should be understood as consistent across time, place and social conditions (Gottfredson and Hirschi, 1990; Hirschi and Gottfredson, 1983). In contrast, others (Farrington, 1986; Greenberg, 1985; Steffensmeier et al., 1989) emphasized variation in the empirical relationship between age and crime over time, between countries and between men and women. For example, data from England and Wales between 1938 and 1983 (Farrington, 1986: 196) showed that men typically had higher conviction rates than women at each age, and with an ACC more concentrated around youth offending, with different patterns of change over time by sex.

Importantly, this debate was not about whether there was *any* variation in the ACC – even Hirschi and Gottfredson accepted that there was empirical change in the ACC over time (1983: 572, footnote 13) – but about whether this observed variation in the ACC was *meaningful*. Meaning could be attributed to observed change in the ACC if this variation helped to understand the causes of the notable correlation between crime and age at the individual level – similar to what Wilson (2012) terms micro-criminology. To some extent, the different perspectives on whether the variation in the ACC was meaningful were a matter of perception (Tittle and Grasmick, 1998: 312) and of semantics (Tittle, 1988) and, given the difficulty of proving whether the ACC should be universally considered as variant or invariant, it is perhaps unsurprising that this debate did not lead to a firm resolution.

### Rediscovering the age–crime curve

The number of recent studies (Blumstein, 2006; Cook and Laub, 2002; Farrell et al., 2015; and Kim et al., 2015) that analysed change in the ACC have very different aims from those conducted as part of the variance/invariance debate. Rather than analysing ACC as a way to understand the individual-level relationship between a person’s age and their propensity to offend, these contemporary studies use variation in the ACC to help unpick trends in *aggregate* crime rates. This is more in line with the concerns of macro-criminology than of micro-criminology (Wilson, 2012). Analysis of the ACC helps to understand macro-level crime trends by assessing if change in crime has been uniform across age or concentrated in particular age groups (Kim et al., 2015). The growing number of recent studies that have examined change in the age distribution of crime are primarily motivated by a desire to understand the recent decline in rates of recorded crime seen in the USA and Western Europe since the early 1990s (Van Dijk and Tseloni, 2012). Put another way, rather than the previous use of the ACC as the *focus of* research, these contemporary studies use the ACC as a *lens for* research.^1^ We can see this distinction between these two interpretations of variation in the ACC in the caution by Loeber and Farrington (2014: 13) – who adhere to more typical micro-criminological concerns – against the analysis of the cross-sectional distribution of age and crime. They contend that cohort ACCs (those for people born in a particular year followed over time) are to be preferred over cross-sectional ACCs (for people of different ages in the same year) to avoid the ‘confounding influence of multiple cohorts’.^2^ But for those interested in using change in the ACC to understand aggregate crime rates it is exactly this kind of cross-sectional analysis and this mix of cohorts that are of interest.

### The crime drop as cohort or period effects

The analysis of change in the ACC is valuable as a way to better understand the crime drop as falls in aggregate crime rates can be conceptualized as comprising period effects or cohort effects (Kim et al., 2015).^3^ In this framework, period effects affect everyone in a given year equally. In contrast, cohort effects affect those born in a particular year regardless of their age. Period effects may not be evenly distributed, affecting those of certain ages (age–period interactions) or from certain birth cohorts (cohort–period interactions).^4^

Kim et al. (2015) use this framework to classify different explanations that have been offered for the crime drop. They consider explanations relating to changes in policing strategies and increased imprisonment rates as period effects, and relative cohort size, the legalization of abortion in the USA and the banning of leaded petrol as cohort effects. To the set of explanations covered by Kim et al. (2015) we can add four other classes of explanations for the crime drop (see Farrell et al., 2015): ‘exogenous’ change in demographics, immigration and drugs markets; change in social norms (Tonry, 2014); better security measures in cars and households, which reduce offending opportunities and prevent young people from beginning criminal careers (Farrell et al., 2014); and changing patterns of routine activities for young people (Aebi and Linde, 2010). As demographics, immigration, drugs markets and routine activities of young people are primarily behavioural explanations they may be more likely to show differential patterns across age than explanations that focus on the operations of the justice system (see Soothill et al., 2004: 415). It is unclear whether changes in social norms are expected to act across all ages or to be more pronounced for different ages, so this explanation may be commensurate with general or age-specific change. Analysing change in the ACC can therefore help refine theories of the crime drop by showing whether explanations should seek to explain change across the age distribution or for particular age groups.

### The age–crime curve and the crime drop

The results of studies into change in the ACC over the crime drop have consistently shown substantial declines in crime rates for young people. Although the methods and data sources used in these studies differ, these studies agree that the crime drop in the USA since the early 1990s was driven by lower rates of offending amongst young people; the crime drop is a *youth* crime drop. This finding is demonstrated by Blumstein (2006), Cook and Laub (2002), Farrell et al. (2015) and Kim et al. (2015), each of whom examine US arrest rates.

These studies consistently described sharp falls in youth arrest rates, despite focusing on trends across very different crime types – homicides between 1985 and 2001 (Blumstein, 2006), violent crime between 1994 and 1999 (Cook and Laub, 2002), change across multiple crime types between 1980 and 2010 (Farrell et al., 2015), and for total arrests between 1990 and 2010 respectively (Kim et al., 2015). Although there have been fewer analyses of change in the ACC outside the USA, some European studies have also shown recent declines in youth convictions, such as Soothill et al. (2008) and Morgan (2014) in England and Wales, Andersen et al. (2016) in Denmark and Bäckman et al. (2014) and Von Hofer (2014) in Sweden. These findings highlight that understanding change in the ACC is an important part of the crime drop.

Patterns of change beyond young adulthood have been less consistent, with different studies showing different trends in arrest or conviction for those in their thirties and older. For example, both Farrell et al. (2015), for violent and property arrests rates, and Kim et al. (2015), for total conviction rates, show increases for those in their late forties in their studies of the crime drop in the USA. However, such increases were not seen by Blumstein (2006), who found declining homicide arrest rates for those in their forties. These contrasting findings provide an indication that different crime types may have shown different patterns of change in their respective ACCs over the crime drop (see Farrell et al., 2015). Moreover, despite international similarities in falling youth crime, findings from the USA for older age groups may not travel well. Morgan (2014: 22) analysed the numbers of people convicted or cautioned in England and Wales between 1995 and 2013 aggregated into 10-year age windows, and found that, whereas the numbers of those aged 10–20 and 21–30 convicted or cautioned have fallen, the numbers of those aged 31 and older receiving these punishments have increased. In Denmark, Andersen et al. (2016) showed that the conviction rate for all age groups declined between 1996 and 2013, but with a greater fall for younger age groups than older ages. However, the use of a single age category for those between ages 31 and 65 by Andersen et al. may obscure potential variation in convictions trends for those over the age of 30, such as those seen by Kim et al. (2015) and Farrell et al. (2015). Taken together, these findings show a strong consensus of falling youth crime but less consistency in trends for older age groups, emphasizing the importance of examining conviction trends disaggregated by age group.

### Limitations of recent studies into the age–crime curve and the crime drop

Although these studies of change in the ACC demonstrate the value of studying variation in this distribution over the course of the crime drop, they have three key limitations. First is their focus on US data. Of the studies identified above that have examined change in the ACC over the crime drop, all except Morgan (2014) and Andersen et al. (2016) focused on US arrest data. This is not a limitation *per se*, but the crime drop has not been experienced in the same way in Europe as in the USA: the US crime drop during the 1990s saw falls in rates of crimes of all crime types; in Europe the crime drop was concentrated in acquisitive crime (Aebi and Linde, 2010). This has led to questions about the capacity to generalize findings from US studies to Europe (Aebi and Linde, 2010) and highlights the value of examining change in the ACC over the course of the crime drop using European data. Moreover, neither Morgan (2014) nor Andersen et al. (2016) examined trends for different types of crime. A more fine-grained analysis of change in the ACC in another European country can provide a useful counterpoint to these existing studies and in turn can help to understand how generalizable trends in USA data are to other countries in Europe other than the UK and Denmark.

A second limitation of these studies is that they have not analysed change in the ACC separately for men and women. Sex differences in the ACC have been noted since its discovery (Quetelet, 2003 [1831]) and are made even more important by the number of recent studies that identified differing recent trends in offending for men and women. In Sweden, both Von Hofer (2014) and Bäckman et al. (2014) compared the conviction patterns of different cohorts born in Sweden between 1958 and 1991 and found declines in the prevalence of convictions in younger cohorts, predominantly driven by declines in the rate of men receiving convictions. Estrada et al. (2016) reported a similar finding also using Swedish convictions data. These findings illustrate that understanding differences between men and women in patterns of change in the ACC over the crime drop are necessary to provide a full account of how the distribution of offending has changed; analysis of ACCs for the population as a whole may obscure different patterns of change between men and women. Moreover, it may be that the declining gender gap in offending as described above may reflect different patterns of change for young men and women and older men and women. Analysing change in the ACC over the course of the crime drop can therefore help to refine our understanding of both these recent trends.

A third limitation of these contemporary accounts of the ACC is that each of these studies examines the ACC using widely dispersed time points (see Appendix 1). The use of disparate time points had been typical in previous analyses of the ACC, and in the context of the ‘great debate’ this focus on widely varying time points was understandable. Hirschi and Gottfredson believed that subtle change in the ACC was meaningless as opposed to the ‘stability of [its] major parameters’ (1983: 572) and for those interested in asserting that the ACC was variant it made sense to focus on maximizing temporal variation in the distribution, to demonstrate that meaningful change was observable over a sufficiently long time span (see Ulmer and Steffensmeier, 2014). However, when the aim is to describe patterns of change in the ACC over the crime drop, this selection of disparate time points entails discarding potentially useful information. Comparing the results of Farrell et al. (2015) and Ulmer and Steffensmeier (2014) provides a useful illustration. Farrell et al. analyse ACCs for homicide in 1980, 1993 and 2010, while Ulmer and Steffensmeier describe the Percentage Age Involvement, an alternative measure of the ACC (see Steffensmeier et al., 1989), for homicide in 1940, 1980 and 2010. Farrell et al.’s (2015) analysis shows an increase in homicide arrests at age 18 from 25 per 100,000 in 1980 to 50 per 100,000 in 1993 and then to 15 per 100,000 in 2010, whereas homicide arrest rates for those aged 25 and above declined between 1980 and 1993. This substantial change is hidden in Ulmer and Steffensmeier’s (2014) analysis because of their selection of time points. By the same principle, analysing annual change in the ACC allows more nuanced trends in the distribution to be identified (Minton et al., 2013). Annual variation is therefore a useful, but previously unused, tool to better understand the patterns of change in the ACC over the crime drop.

## Methods

### Data

Analysing change in the ACC requires consistently collected data across a wide range of ages and over time. This typically involves using administrative data (Steffensmeier et al., 1989) where it is difficult to separate the effects of offending and of official reactions to offending, and consequently such data are best understood as representing official reactions to offending behaviour, rather than offending behaviour itself (Francis et al., 2004a: 54). Whilst it would be desirable to triangulate across different types of data sources, the capacity to do so is limited by data availability.

#### Data source

The Scottish Offenders Index (SOI) provides a previously unexamined source by which to investigate change in the ACC over the recent crime drop. The version of SOI used for this analysis contains a census of conviction proceedings served in adult courts in Scotland between 1 January 1989 and 31 July 2013, with the exception of more minor criminal convictions such as some crimes against the court and motoring offences.^5^ SOI holds information about offenders’ age and sex and the date of their offence, as well as the details of their conviction. In Scotland, children under the age of 16 are handled by a separate youth justice system (McAra and McVie, 2015), although it is possible for people below this age to receive convictions in adult courts. As SOI covers court convictions, the data contain a reliable estimate of convictions only for those over the age of 16 (Scottish Government, personal communication, 2014).

**Figure 2.**
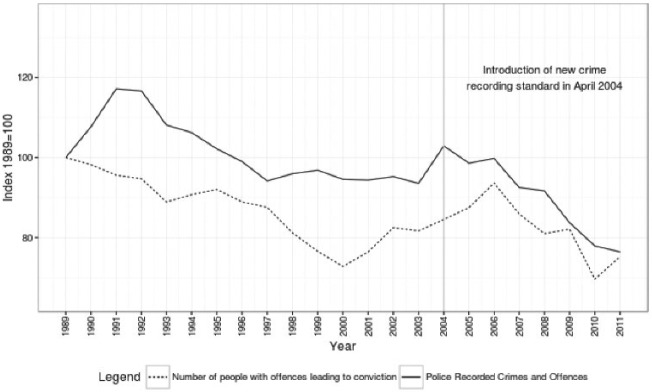
Trends in police recorded crime and convictions in Scotland, 1989–2011.

The suitability of SOI as a data source for this analysis would be moot if Scotland had not experienced a crime drop. Figure 2 shows that Scotland has seen a crime drop since the early 1990s, as well as declines in the total number of convictions served in Scottish courts.^6^ The Scottish Crime and Justice Survey also shows declines in victimization in Scotland between 2008 and 2013 (McVie et al., 2014). Scottish Government statistics (2016) show that the fall in recorded crime in Scotland is primarily concentrated in acquisitive crime, similar to Aebi and Linde’s (2010) account of the crime drop in Europe. Although some differences were noted between Scotland and other countries in Western Europe,^7^ Aebi and Linde’s (2012) analysis of conviction trends in Europe between 1990 and 2006 suggests that trends in Scotland are more likely to be similar to other countries in Western Europe than to those in Central and Eastern Europe. Such factors make examining changes in the age–crime curve over time in Scotland using SOI a useful way to further the analysis of recent change in the ACC over the course of the crime drop in Western Europe.

### Measures

For this study the ACC is defined as the age-specific rates of convicted offences per 10,000 population between ages 16 and 60,^8^ with the person’s age measured at the date of offence. Results are presented for men and women separately.^9^ Age-specific rates are presented to account for population change over time based on the mid-year population estimates for each age in each year (National Records of Scotland, 2014). One consequence of focusing on age at the time of offence is that it required the exclusion of offences committed in 2012. As SOI extends eight months after the end of 2012, a number of offences committed in 2012 are likely to still be processing through the justice system by the end of July 2013.^10^ This makes the rates of convicted offending for 2012 less comparable with convicted offending rates in earlier years, and so limiting the scope of the analysis to 2011 allows a more valid comparison across the SOI.

Figures are presented for total convictions and then disaggregated by crime type, split by crimes of dishonesty, violence and other crimes. These classifications are based on those used by the Scottish Government, and distinguish forms of theft and acquisitive crime (included in the dishonesty group) from violent crime and other kinds of crime. Appendix 2 lists the offences included in these groups. These categories were selected because of the substantive importance of the distinct trends between acquisitive and violent crime seen over the course of the crime drop in Europe (Aebi and Linde, 2012).

### Analytical approach

The primary method of analysis is visual inspection of distributions of age and crime (Tittle and Grasmick, 1998). This visual analysis is supplemented by descriptive statistics.

#### Visual analysis

We use two methods to visualize change in the ACC: shaded contour plots (Minton et al., 2013; Vaupel et al., 1987) and line charts. Shaded contour plots provide a way to analyse changes in a particular variable (*z*) across age (*x*) and year (*y*). By arranging age and year as a surface, the dependent variable (*z*) – in this case, the prevalence of convicted offending – for a particular age in a particular year can be read as the ‘height’ of the surface. Change in height across this surface can be shown as contours linking similar values together, and, as on a topographic map, these contours can be labelled to show the values that are connected and the surfaces can be shaded to help distinguish between high and low values (Minton, 2014). This facilitates comparison of data across multiple years in the same chart and allows the informal examination of age, period and cohort effects, allowing assessment of whether different periods show different patterns of change in the ACC. Line charts are used to emphasize key trends by showing the average ACC for different periods identified using the shaded contour plots.

**Figure 3.**
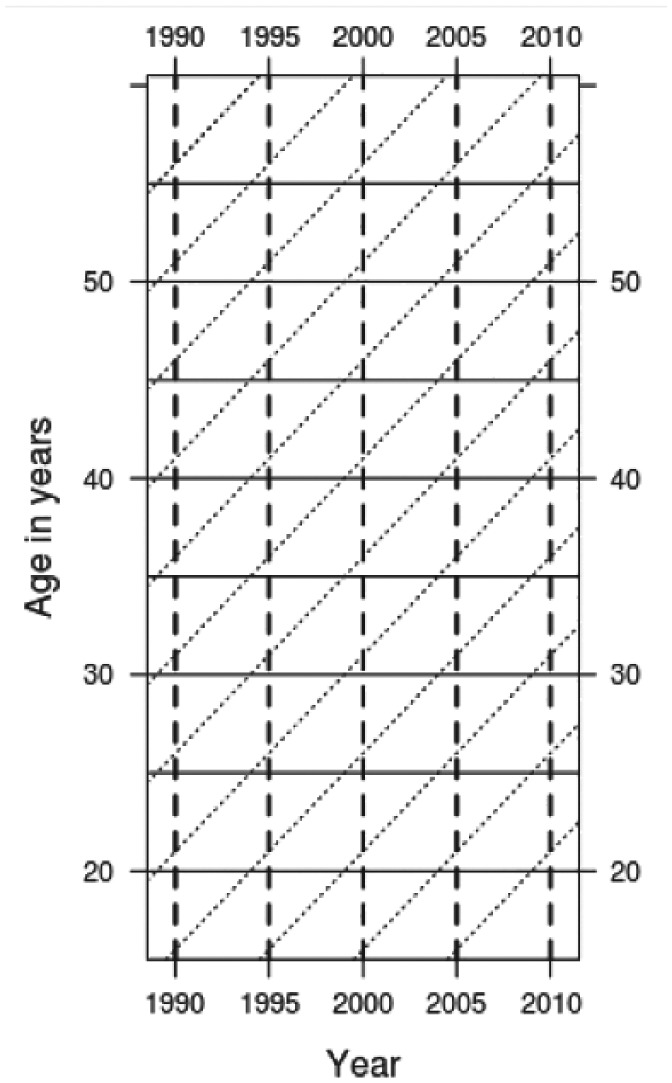
Identifying age, period and cohort effects from a shaded contour plot.

Figure 3 shows how to distinguish between age, period and cohort effects within a shaded contour plot. With year running across the horizontal axis and age running across the vertical axis, period effects are identifiable as changes in the surface of cells when viewing the image from left to right or right to left (the solid horizontal lines in Figure 3), age effects as changes in the surface when viewing bottom to top or top to bottom (dashed vertical lines), and cohort effects are changes that occur primarily along the diagonal (dotted) lines. If there was no change over time in the ACC we would expect to see only horizontal contours, and so deviations from horizontal lines in the contour plots illustrate change in the distribution of age and convicted offending.

#### Descriptive statistics

To supplement the visual analysis we also measure change in a number of descriptive statistics: the mean, median and modal age of conviction, the skew and kurtosis of the distribution, and the convicted offending rate at the peak age. These measures are recommended by Farrington (1986) as ways to measure change in the ACC. Mean, median and mode give an indication of where the centre of the distribution is. Skewness measures how symmetrical a distribution is, with positive values showing a distribution with a longer right-tail, and more mass concentrated on the left-hand side of the distribution. The typical ACC is positively skewed (Farrington, 1986). This is likely to be exacerbated in SOI because the data begin at age 16 and so the distribution’s left-tail is artificially truncated. Kurtosis measures how much a distribution is peaked around its centre. The more the mass of the distribution is concentrated around the peak value the higher the kurtosis. Kurtosis is measured relative to the Normal distribution (with mean zero and standard deviation of one). Here a kurtosis value of zero is the amount of peakedness present in a normal distribution.

## Results

### Change across age and sex

**Figure 4.**
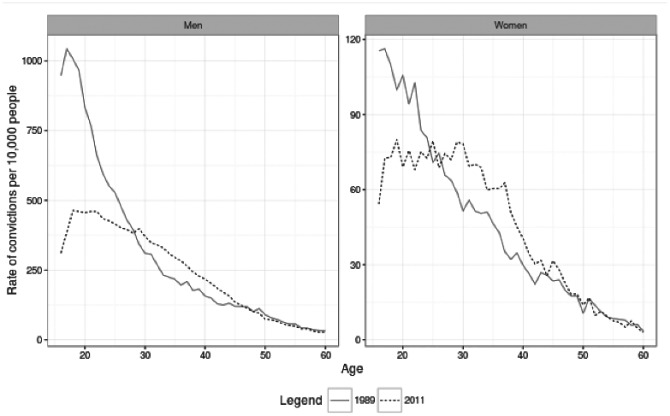
Age–crime curves in the SOI, 1989 and 2011.

Figure 4 demonstrates that over the period covered by the SOI both men and women in their mid-twenties and younger have seen declines in rates of convicted offending, whereas those between their late twenties and mid-forties have seen rates of convicted offending *increase*. For both men and women, we see that there is a disparity in declining rates of convicted offending, with declines being concentrated amongst those aged 25 and below. However, the relative magnitude of these changes differs by sex; the drop in convicted offending is greater for young men than for young women, and the relative increase is greater for older women than for older men. This can be seen in Table 1, which compares the magnitude of change in convicted offending rates for men and women aged 17 and 30 between 1989 and 2011. Young women show less of a decline in convictions over the course of the crime drop than young men, and older women show greater increases in convictions during this period than older men.

**Table 1.** Comparison of change in rates of convicted offending for men and women aged 17 and 30 in 1989 and 2011.

Age	Sex	Convicted offending rate Year		Relative change (percent)
		1989	2011	
17	Men	1043	385	−63
	Women	116	73	−37
30	Men	310	374	21
	Women	51	78	53

*Source*: SOI.

The result of these divergent trends is that the shape of the ACC for men and women is more similar in 2011 than it was in 1989, illustrated by the descriptive statistics for ACCs for men and women in 1989 and 2011 presented in Table 2. For both sexes we see increases in the mean and median ages of offending, but much less change in the modal age of offending. Thus, whereas the typical age of those with convicted offences increased, the location of the peak of the ACC did not. There are also falls in the convicted offending rate at peak age for men (dropping by just over 65 percent) and more modest declines for women (declining by just over 31 percent), combined with declines in skewness and kurtosis. Together these results describe a ‘flatter’ ACC for both men and women in 2011 than in 1989. Because the rate of convicted offending at the peak age has declined, the ACC has a less distinctive peak in 2011 than 1989.

**Table 2.** Descriptive statistics for age–crime curves in 1989 and 2011 split by sex.

Measure	Year					
	1989			2011		
	Men	Women	Difference	Men	Women	Difference
Mean	26.2	28	*–1.8*	30.8	31.3	*–0.50*
Median	23	25	*–2*	29	30	*–1*
Mode	17	17	*0*	18	19	*–1*
Skew	1.8	1.2	*0.6*	0.7	0.6	*0.1*
Kurtosis	2.4	0.3	*2.1*	−1.1	−1.3	*0.2*
Convicted offending rate at peak age	1043.4	116.3	*927.1*	463.7	80.0	*383.7*

*Source*: SOI.

*Notes*: Figures to 1dp. Rates are per 10,000 population.

Comparing figures for skew and kurtosis, we can see that there has been more change in kurtosis than skew for both men and women. This reinforces that most of the change in the distribution of age and crime relates to the size of the peak (kurtosis) rather than where in the age distribution the peak is (skew), echoing the findings of increasing mean and median age of conviction but a relatively stable modal age. Consequently the peak of the ACC is much less pronounced for both men and women in 2011 than in 1989. In 1989 the peak of the ACC in the late teens marked a period of distinctively high rates of convicted offending, and whilst the age at which convicted offending peaks is similar in 2011 rates of convicted offending in the late teens are very similar to those for people through their twenties.

### Trends across time

**Figure 5.**
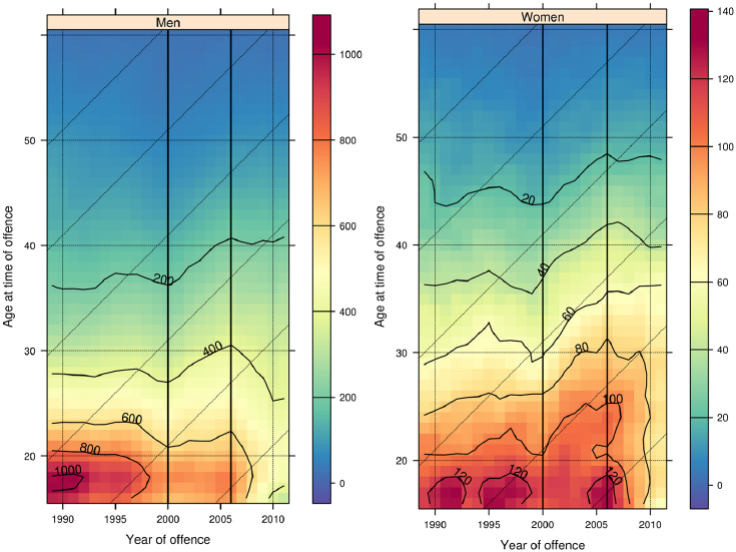
Shaded contour plot of convicted offences in the SOI, 1989–2011.

So far this analysis has focused on comparing the start and the end of the data covered by SOI. Figure 5 shows a shaded contour plot showing the age-specific conviction rates for each single age from 16 to 60 years, and each year from 1989 to 2011. Year runs left to right on the *x* axis, and age runs low to high on the *y* axis. The colours indicate each of the individual age-, year- and sex-specific offending rates (per 10,000 population), based on the scales presented to the right of each sub-figure (colour figures available online). Contour lines link together positions across the contour plot of equal height, labelled with their corresponding values. Contours mark a change of 100 convicted offences per 10,000 population on the sub-figure for men and 20 convicted offences per 10,000 for women.

Looking top to bottom we can see that contour lines are mostly horizontal, showing the typical age effect that would be expected from the ACC. Looking left to right shows that the overall change in the ACC seen in Figure 4 does not occur linearly between 1989 and 2011. Instead, the dynamics of change form three reasonably distinct periods of change in the data between 1989 and 2000, 2001 and 2006 and 2007 and 2011, marked on the contour plot by dashed vertical lines.

These periods are marked by different trends in convicted offending between those in their mid-twenties and younger, and those from their mid-twenties to their early forties. The period 1989–2000 shows a marked decline in rates of convicted offending for young men. We can see this in the contour plot by looking at the peaks of convictions centred on the years 1991, 1996 and 2001 around age 18. The highest contours in these peaks show convicted offending rates of 1000, 950 and 800 (not labelled), indicating a decline in rates of convicted offending in the late teens between 1989 and 2000. Comparing these peaks with the equivalent ages and years for women we see that the convicted offending rates at these ages are close to 130, 130 and 120 respectively; whereas convicted offending rates for young men dropped by almost a quarter during the 1990s, the convicted offending rate for young women showed very little change. In contrast to these declines in convictions for young men, over this period the contour lines for those aged 22 and above show little change for both men and women. The period between 1991 and 2000 also saw declines in recorded crime (Figure 2), and so Figure 5 shows that this crime drop in Scotland in the 1990s is associated with declining rates of convicted offending for young men but not for young women or for older adults.

Between 2001 and 2006, however, there is a distinct change in trends of convicted offending rates. First, the decline in convictions for young men ceases. The highest contour in 2006 for men is labelled 800, the same value as the contour in 2001. There is also little change in conviction rates for young women during this period, and indeed by 2006 there has been very little decline in rates of convicted offending by young women since 1989. However, convicted offending rates for men and women in their mid-twenties to early forties *increase*. We can see this in the diagonal trend in the contour lines for both men and women over the age of 20 after 2000. For example, for men aged 30 in 2000 the nearest contour line shows a convicted offending rate of 300, but by 2006 the closest contour line for 30-year-old men is for 400 convictions. The overall change that we see in convicted offences for men and women in their mid-twenties to forties in Figure 4 occurs entirely in this period. The increases in convicted offending rates across age starting at the same time suggest a period effect, with the diagonal contour lines caused by period effects interacting with the strong age effect of the ACC.

The final period spans from 2007 until 2011 and is marked by rapid declines in convicted offending rates for both men and women under 20 but stable rates of convicted offending for ages 30 and above. This is shown by the concentration of contour lines in the bottom right of the plot for men and the sharp vertical turn in the contour line labelled 100 for women. Compared with the initial period of declining convicted offending rates during the 1990s, this period shows an even greater magnitude of decline in convicted offending rates for young men. From the highest contour of convictions at age 18 in 1991, the rate declines to between 70 and 80 percent of its peak in 2000, a drop of between 250 and 300 convictions per 10,000. Between 2006 and 2011 – a much shorter time span – convicted offending rates for 18-year-old men decline from between 800 to 850 to between 450 and 500; a decline of a greater magnitude in a shorter period of time. Taking the same years as a comparison, the drop is even more marked for women. Figure 5 shows a decline from a convicted offending rate close to 130 for women aged 16 in 2006 to a lowest contour of 60 (not labelled) for women aged 16 in 2011. Almost all of the declines in the ACC that we see for young women in Figure 4 occur in this period. In contrast to this decline for young people, rates of convicted offending for both men and women over 30 remain close to their rates in 2006, shown by the mostly horizontal contours above this age. This final period is also marked by declines in police recorded crime, total numbers of convictions (see Figure 2) and victimization (McVie et al., 2014). Unlike the crime drop in the 1990s, this period of declining crime is associated with declines in convicted offending rates for both young men and young women. However, like the first period there is little change in convicted offending rates for those in their mid-twenties and above.

### Trends across crime type

**Figure 6.**
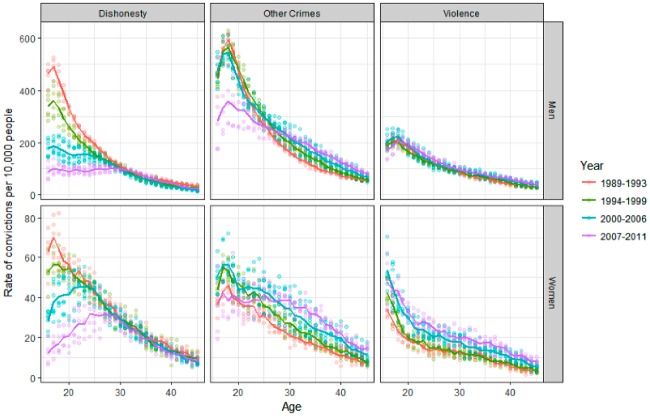
Age–crime curves in the SOI for violence, dishonesty and other crimes, 1989–2011.

**Figure 7.**
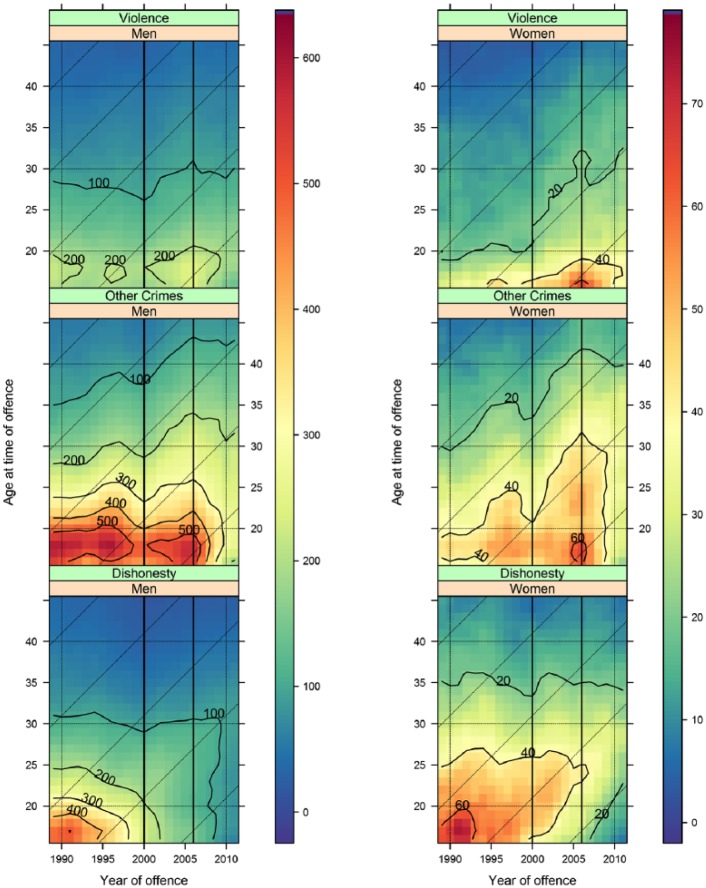
Shaded contour plot of convicted offences of different crime types in the SOI, 1989–2011.

Figure 6 presents ACCs for three crime types: dishonesty, violence and other crimes. In this figure age runs from left to right, with the annual prevalence rate on the *y* axis. The three plots on the top row show figures for men, and those on the bottom row show figures for women. The solid lines represent average ACCs for different time periods, with year-specific prevalence rates represented by coloured points. Drawing on the results presented so far, the periods identified in Figure 5 are represented in different colours in Figure 6. The first period is shown in red and green, the second period in blue and the third period in purple. Given space limitations, this visualization highlights the key trends in the data, as shown by the solid lines, as well as indicating the annual variation in the distribution. The same data are represented in a shaded contour plot in Appendix 3 (Figure 7).

Figure 6 illustrates three main findings. First, it demonstrates that different types of crime show different patterns of change in their respective ACCs. Second, these patterns of change are different for men and women. Third, Figure 6 shows that the three different types of crime show different schedules of change.

Looking first at trends for men, the most striking trend in Figure 6 is that, for men under the age of 30, convictions for crimes of dishonesty decline steadily from 1989 to 2011, but for men above this age there is very little change in the prevalence of convictions for dishonesty. In contrast, the ACC for violent convictions shows much less change over time, although there is a fall of around 30 percent in the prevalence of violent convictions for teenage men between 2007 and 2011. Convictions for other crime types show a steep drop for men under the age of 25, but also only after 2007. However, convictions for other crime types also *increase* for men above the age of 30 in each successive time period. Taking these results together, Figure 6 suggests the first period of overall decline in convictions for young men through the 1990s is driven primarily by a decline in convictions for crimes of dishonesty. Although convictions for dishonesty served to young men also decline after 2007, the fall in overall convictions during this period is a more general effect spread across all three crime types analysed. In contrast, the increase in convictions from 2000 to 2007 for those in their mid-twenties to late thirties is primarily the result of increases in convictions for other crime types, with only small increases in the prevalence of violent convictions and little change in the prevalence of convictions for dishonesty.

In one respect the results for young women are similar; young women also show declines in the prevalence of dishonesty in each successive period, although the falls during the 1990s are less pronounced for young women than they are for young men. The prevalence rate for dishonesty in 2000 for men aged 17 was around 45 percent of the 1989 rate; for women aged 17 in 2000 it was around 75 percent of the 1989 rate. However, women of all ages show higher prevalence of convictions for violence in each consecutive period. These increases are relatively larger for women than for men. Taking those aged 17 as an example, the prevalence rate for violent convictions for men in 2000 was around 85 percent of its 1989 levels, whereas for women of this age the rate had increased to around 145 percent of the 1989 value. By 2007 the prevalence rate for violence for 17-year-old men had returned to almost exactly the same value as the 1989 rate, whereas for 17-year-old women the prevalence rate was approximately 185 percent of its 1989 rate. In the last period, however, there is a decline in the prevalence of violent convictions for women in their late teens between 2007 and 2011, similar to the trend seen for men.

The prevalence of convicted offending for other crimes also increases for women of all ages between 1989 and 2007, although after this point there is a sharp decline in the prevalence of conviction for other crimes for women below the age of 30. Again, this decline in convictions for other crime types seen for young women is similar to the trend seen for young men.^11^

Drawing these results together, Figure 6 shows that the stability of overall convictions for young women during the 1990s seen in Figure 5 actually represents the net effect of the lower prevalence of convictions for dishonesty offsetting increases in convictions for violence and for other crime types. Overall, Figure 6 adds nuance to the results presented in Figure 5, illustrating that the first period of crime drop during the 1990s was one concentrated in a falling prevalence of convictions for dishonesty that affected only young people, and was seen more for young men than for young women. The increases in convictions for those in their mid-twenties to forties between 2000 and 2007 is a more general effect concentrated in other crime types and violent convictions, with women particularly showing increases in violent convictions. The second period of crime drop from 2007 to 2011 is a more general phenomenon than the first, with falls in the prevalence of convictions for young people across all three crime types analysed.

## Discussion

The results presented above show substantial change in the aggregate ACC in Scotland between 1989 and 2011, becoming flatter as overall conviction levels fell, and provide a number of insights regarding the development of the crime drop in Scotland.

### The crime drop in Scotland is a youth crime drop

The finding of a substantial decrease in convicted offending rates for young people over the course of the crime drop is consistent with the findings from other countries, reinforcing the conclusion that the crime drop is primarily a youth crime drop. However, increases in convictions for those in their mid-twenties contrast with results from the USA, which did not see increases in arrest rates for older adults over the course of the crime drop until their early forties (Farrell et al., 2015; Kim et al., 2015). In Scotland, overall falls in conviction rates are driven by declines in convicted offending for young people, which obscure increases in convicted offending for adults from their mid-twenties to their forties.

These discrepancies may reflect differences between the manifestation in the crime drop in the USA and Scotland, or between convictions and arrest data, or both. Given its descriptive focus, this study alone cannot explain this increase. The increase in convictions for those in their mid-twenties to forties in Scotland is similar to trends observed in England and Wales (Morgan, 2014) but not in Denmark (Andersen et al., 2016), although it should be noted that the different methods adopted in this study and by Morgan and Andersen et al. limit the capacity for direct comparison. Nevertheless, although the finding of the youth crime drop is consistent across jurisdictions, trends for older adults may be less generalizable. We encourage replication of this analysis and examining rates of convictions across the whole age distribution to further investigate this issue.

### The crime drop is manifest differently in patterns of convicted offending for men and women

The results reinforce the value of analysing change in the ACC for men and women separately by showing that declines in offending over the crime drop have been disproportionately experienced by men (Bäckman et al., 2014; Lauritsen et al., 2009; Von Hofer, 2014). The discrepancy in convictions patterns between older and younger men and women suggests that to analyse changes in the gender gap without accounting for age, and vice versa, can give only a partial picture of change in aggregate crime rates. The conclusion of Lauritsen et al. (2009: 391) that, ‘whatever social forces have reduced offending over the crime drop, they have not benefited women and men equally’ should be extended to cover differences between young and old as well.

### The crime drop in Scotland has not been a linear process

The two periods of declining recorded crime through the 1990s and from the mid-2000s are represented by very different patterns of convictions. The overall drop in convictions during the 1990s is associated with declining convicted offences for young men. These overall trends reflect a greater decline in the prevalence of convictions for crimes of dishonesty for young men as compared with young women, and increases in convictions for other crime types seen for young women but not for young men. In contrast, the more recent decline in crime from 2007 is reflected in falling convicted offending rates for both young men and young women and is seen across the three crime types analysed. These non-linear patterns of change in convicted offending raise the possibility of different mechanisms leading to different ‘periods’ of the crime drop, or suggest that the same mechanism was manifest in very different patterns of change at different times. The discrepancies seen in the magnitude of change across different crime types in different periods reinforce this conclusion. This periodization emphasizes that, with a descriptive and empirical focus, annual change in the ACC becomes a useful tool by which to understand change in aggregate crime rates. The different trends observed between 1989 and 2000, 2001 and 2006 and 2007 and 2011 would be obscured if this analysis had relied on the examination of widely dispersed time points. This kind of close reading of the available data, made possible using shaded contour plots, provides valuable information for those wishing to theorize about the causes of the crime drop and in particular provides a tool to identify different periods in the data that may indicate period effects affecting conviction rates.

For Scotland particularly, these results are important for those interested in the recent development of the justice system. Although the correlation is not perfect, there is a notable similarity between the trends identified in these conviction patterns and policy ‘eras’ in Scotland. In particular, the increase in convicted offending rates in the early 2000s aligns with the ‘punitive turn’ in the discourses around youth crime and antisocial behaviour in Scotland between 1999 and 2006, and declines in youth convictions after 2007 align with a period of ‘compassionate justice’ in Scotland (McAra, 2017). Because conviction statistics are social constructs that are necessarily influenced by changes in justice system practices (Francis et al., 2004b: 123), this potential association between changes in policy and the observed changes in convicted offending is an area that would benefit from further investigation.

Moreover, the different trends seen across different types of crime, and particularly between violence and crimes of dishonesty, contrast with similar patterns of change by age seen for different crime types in US arrest rates (Farrell et al., 2015). This observation reinforces the point made by Aebi and Linde (2010) that the crime drop is not the same in Europe as in the USA. We encourage further replication of this analysis to establish whether the trends identified here are typical of the crime drop in Western Europe more generally. We argue that shaded contour plots provide a simple method by which to present large quantities of data and so facilitate comparisons across age, sex and crime type, helping to prevent erroneous conclusions being drawn from the coarse aggregation of conviction rates into wide age or year groups.

### Theories of the crime drop must be able to account for these patterns

These findings reinforce the claim that the mechanism driving declining crime rates is not a period effect acting upon all members of society equally (Kim et al., 2015) and suggest that the crime drop is likely to be a cohort effect, age–period interaction or cohort–period interaction. This likely excludes possibilities based on policing or imprisonment (Kim et al., 2015). Substantively, these findings are similar to those of Kim et al., whose analysis suggests a cohort explanation for the crime drop and who also note an age–period interaction for the youngest cohorts in their analysis, suggesting that different age groups show different patterns of change over time. Although these observations are compatible with a number of explanations for the crime drop, it may be helpful for theories of the crime drop to be specified explicitly in terms of age, period and cohort effects. Moreover, based on the observation of different trends of decline in rates of convicted offending for men and women and over time, accounts of the crime drop must also explicitly account for sex differences when explaining why crime has fallen. For example, contrasting patterns across age and sex could be added to Farrell’s (2013) ‘varying trajectories’ test, which specifies that theories of the crime drop must be able to account for differing patterns of decline between countries.

However, it may also be that the framework of looking for a *single* theory of the crime drop (see Farrell, 2013) that can be reconciled with divergent trends across different crime types in different times may lead to conclusions that are too broad. Instead, what might be required is an understanding of the particular factors affecting falling crime rates in different periods (Humphreys et al., 2014). Indeed, Farrell et al. (2014: 457) provide an example of such a synthesis, suggesting that wider Internet access through the mid-to-late 2000s may have had a ‘consolidation effect’ in further reducing crime rates after initial reductions owing to their favoured securitization hypothesis. The results presented here are consistent with, but should not be read as proof of, this assertion. However, our results do suggest that more precisely specifying how and why hypothesized mechanisms that have led to the crime drop have acted across age, sex, time and crime type is an important avenue for our understanding of the crime drop. The different results observed between 1989–2000 and 2007–11 suggest that explanations of the crime drop based on data from the 1990s may not be generalizable to current crime trends.

### The age–crime curve in Scotland has shown substantively meaningful change over the crime drop

Whilst the aims of this study are different from those who analysed change in the ACC as part of the variance/invariance debate, the scale of the change in the ACC demonstrated here is more in keeping with a perspective that emphasizes the empirical variation in the relationship between age and crime at the aggregate level (for example, Farrington, 1986) and that treats annual variation in the ACC as a subject worthy of study. Loeber and Farrington’s (2014) description of the curve as peaking in the teenage years and declining from the late teens or early twenties is still technically accurate, but the ‘peak’ of the aggregate ACC in Scotland is now almost flat. Although it may be that the causal effect of age and crime was the same in 2011 as in 1989 (Gottfredson and Hirschi, 1990), if so, the way this effect was manifest in aggregate patterns of convicted offending in 2011 is very different than in 1989. For those interested in examining the effect of age on crime, the task has shifted from explaining an increasingly peaked ACC (for example, Moffitt, 1993: 691–2) to explaining an increasingly flat distribution. This analysis demonstrates the value of current and ongoing analysis of the aggregate ACC, but also of adopting a different conceptual approach to the observation of variation in the ACC from the participants in the ‘great debate’ and their focus on variation across widely dispersed time points.

### Limitations

Our research is not without limitations. First, in focusing only on sex and age our study has not considered other potentially important factors related to the shape of the ACC, such as socio-economic status. This has been shown to be an important factor in understanding the crime drop (Estrada et al., 2016). Such data are not currently available in Scotland and were therefore not included in this analysis. Second, owing to the nature of the data held in the SOI we have been unable to examine changes in offending for those under the age of 16. This limits the capacity of our findings to understand changes in the period typically associated with mass onset of offending during early adolescence (Moffitt, 1993) and is another area that future research may benefit from exploring.

## Conclusions

The analysis presented in this paper has extended the literature using variation in the ACC to understand the development of the crime drop. This analysis makes three key contributions to this literature: by analysing data in a previously unexamined country; by considering trends separately for men and for women; and by analysing annual variation. Declining youth crime is common across European and US studies, but increases in convicted offending for adults between their mid-twenties and early forties identified in Scotland had not been seen in previous US analysis. The findings of US studies are therefore not generalizable to other contexts without modification, highlighting the importance of international comparison and updating analyses over time to understanding the crime drop. Examining change for men and women separately has shown similar overall patterns of change in convicted offences for men and women between 1989 and 2011, but of differing magnitudes and differing timings. Analysis split by crime type has shown further differences in patterns of change between men and women. These findings highlight the value of accounting for both age and gender when analysing change in conviction patterns over time and help to reconcile the previous findings of both declining youth crime and a declining gender gap in offending. Finally, the results presented here show the value of empirically assessing annual change in the ACC, demonstrating that change in the ACC over the crime drop has been not a linear process but rather one that seems to have operated in distinct periods. At this stage quite how to interpret these periods is unclear, but this observation can act as a useful starting point for future studies seeking to unpick recent changes in crime rates both in Scotland and elsewhere. The results presented here demonstrate that to properly understand the crime drop we need to empirically explore – and be able to explain – different trends across age, sex, crime type and different periods.

## Appendix 1

**Table 3.** Studies investigating change in the age–crime curve and time points analysed.

Study	Case	Time points
Blumstein (2006)	USA	1985, 1993, 2001
Farrell et al. (2015)	USA	Initial year of 1980 and final year of 2010. Middle year varied by crime type between 1988 and 1994
Farrington (1986)	England and Wales	1950, 1960, 1970 and 1980, 1938, 1961 and 1983 for men and women separately
Greenberg (1985)	USA	1970, 1975, 1980
Hirschi and Gottfredson (1983)	England and Wales USA	1842–1844, 1965 (England and Wales), 1977 (USA)
Kim et al. (2015)	New York State, USA	1990–2010 five-year increments
Steffensmeier et al. (1989)	USA	1940, 1960 and 1980
Ulmer and Steffensmeier (2014)	USA	1940, 1980 and 2010

## Appendix 2

**Table 4.** Crime types included in the analysis.

Crime type	Offences include
Violent crime	Murder, culpable homicide, attempted murder, serious assault, robbery, common assault, death involving a motor vehicle, other violence
Dishonesty	Housebreaking, theft by opening lockfast places, theft of motor vehicle, other theft, fraud, other crimes of dishonesty and social security offences
Other crimes	Fire-raising, vandalism, illegal importation, supply or possession of drugs, other drug offences, breach of the peace, racially aggravated harassment, racially aggravated conduct, threatening or abusive behaviour, offence of stalking, offensive behaviour at football, and threatening communications (under the Offensive Behaviour at Football and Threatening Communication Scotland Act 2012), crimes against public justice (breach of sexual offender order and breach of sexual harm order are included in crimes against public justice), handling offensive weapons (in possession of an offensive weapon; having in a public place an article with a blade or point, and restriction of weapons), miscellaneous firearm offences, other crimes and offences (not elsewhere specified), sexual crime and prostitution

## Notes

1. Or ‘data signature’ in Farrell et al.’s terminology (2015: 7).

2. There may also be a disciplinary element to this difference, with differing understandings of age coming from those in the tradition of developmental psychology (Loeber and Farrington, 2014) and demography (for example, Kim et al., 2015).

3. The contention that change in the age–crime curve reflects different mixes of age, period and cohort effects is not new (Farrington, 1986: 205); what is novel is the application of this framework to understand recent declines in overall crime rates.

4. Age–period interactions are indistinguishable from trends in cohort effects (Cook and Laub, 2002: 25, footnote 18), and so most simply the distinction of interest is between changes that are experienced equally across age in a given time point (period effects) and that are seen by only some age groups (cohort effects, age–period interactions or cohort–period interactions).

5. Because of the volume of these less serious crimes, it is estimated that SOI covers between 52 percent and 58 percent of the total number of convictions in Scottish courts. Of this discrepancy, between 79 percent and 86 percent is due to motor vehicle offences. Calculations available on request.

6. Recorded crime in Scotland was collected per calendar year until 1995, after which it was collected 1 April to 31 March. Figures marked as 1995 reflect 1 April 1995 – 31 March 1996, and so on. Figures for convictions represent calendar years throughout. Recorded crime figures are presented for recorded crime types comparable to SOI crimes, and so exclude motor vehicle offences and crimes against public justice.

7. For example, Aebi and Linde (2012) show that Scotland was one of 11 countries to see increases in convictions for assault between 1990 and 2006, but only Scotland and Northern Ireland had lower rates for robbery in 2006 than in 1990. Still, and although this was not the focus of their analysis, Aebi and Linde do not identify conviction trends in Scotland as being a particular outlier amongst West European countries.

8. Analysis is capped at age 60 owing to the low rates of convictions for people over this age.

9. Because the prevalence of conviction is much higher for men than for women, trends for the overall prevalence of convictions are essentially the same trends as those for men.

10. We estimate that the follow-up period to 31 July 2013 leads to an underestimate of the total number of convicted offences in 2012 by around 12.5 percent, compared with an undercount of 1.5 percent of convicted offences in 2011. Calculations available on request. In addition, based on exploratory analysis not presented here, the fall in prevalence seen across all ages in 2010 represents a data quality issue rather than a sudden change between 2009 and 2010. That no similar change is seen in the Scottish Government’s (2017) figures for criminal proceedings supports this interpretation. As such, this decline in prevalence in 2010 should not be interpreted substantively.

11. From the diagonal lines seen in the contour plot for ‘Other Crimes’ presented in Appendix 3 (Figure 7) for both men (labelled 200) and women (labelled 30), these effects are most plausibly seen as a cohort effect.
